# Melatonin Is a Potential Target for Improving Post-Harvest Preservation of Fruits and Vegetables

**DOI:** 10.3389/fpls.2019.01388

**Published:** 2019-10-30

**Authors:** Tao Xu, Yao Chen, Hunseung Kang

**Affiliations:** ^1^Key Lab of Phylogeny and Comparative Genomics of the Jiangsu Province, Institute of Integrative Plant Biology, School of Life Sciences, Jiangsu Normal University, Xuzhou, China; ^2^Department of Organismic and Evolutionary Biology, Harvard University, Cambridge, MA, United States; ^3^Department of Applied Biology, College of Agriculture and Life Sciences, Chonnam National University, Gwangju, South Korea

**Keywords:** melatonin, fruits, vegetables, post-harvest preservation, ripening

## Abstract

Melatonin is a ubiquitous molecule distributed in nature and not only plays an important role in animals and humans but also has extensive functions in plants, such as delaying senescence, exerting antioxidant effects, regulating growth and development, and facilitating plant adaption to stress conditions. Endogenous melatonin is widespread in fruits and vegetables and plays prominent roles in the ripening and post-harvest process of fruits and vegetables. Exogenous application of melatonin removes excess reactive oxygen species from post-harvest fruits and vegetables by increasing antioxidant enzymes, non-enzymatic antioxidants, and enzymes related to oxidized protein repair. Moreover, exogenous application of melatonin can increase endogenous melatonin to augment its effects on various physiological processes. Many previous reports have demonstrated that application of exogenous melatonin improves the post-harvest preservation of fruits and vegetables. Although overproduction of melatonin in plants via transgenic approaches could be a potential means for improving the post-harvest preservation of fruits and vegetables, efforts to increase endogenous melatonin in plants are limited. In this review, we summarize the recent progress revealing the role and action mechanisms of melatonin in post-harvest fruits and vegetables and provide future directions for the utilization of melatonin to improve the post-harvest preservation of fruits and vegetables.

## Introduction

Fruit and vegetable senescence is an irreversible process in nature that involves a series of physiological, biochemical, and metabolic changes, which is accompanied by a decline in color, flavor and nutrition, and ultimately shortening the shelf life (Prasanna et al., 2007; Rugkong et al., 2011). The post-harvest decay of fruits and vegetables is mainly due to the continuous consumption of their own nutrients through respiration resulting in chlorophyll destruction and decomposition, substrate oxidation, cell wall softening, and membrane penetration; their nutritional value constantly changes under the influence of temperature, humidity, and air composition (Bureau et al., 2006; Barrett and Lloyd, 2012). Therefore, to improve post-harvest preservation, many traditional physical storage methods such as refrigeration, controlled atmosphere storage, and ventilation storage have been discovered for prolonging the storage period of fruits and vegetables (Zhou et al., 2000; Adeyeye et al., 2017). With the rapid development of biotechnology, some chemical reagents have also been utilized to prolong the shelf life of fruits and vegetables (Kuang et al., 2008; Wang et al., 2012).

Melatonin is a hormone secreted by the pineal gland in animals. It was first isolated in 1958 in humans and was discovered in the family of monocotyledonous and dicotyledonous edible plants in 1995 (Dubbels et al., 1995; Hattori et al., 1995). Melatonin is synthesized from tryptophan through the catalysis of tryptophan decarboxylase, ryptamine-5 hydroxylase, 5-hydroxytryptamine-N-acetyltransferase, and N-acetyl-5 hydroxytryptamine-methyl transferase in plants, which are then catabolized to 2-hydroxymelatonin by the action of melatonin 2-hydroxylase (Rui et al., 2016). It is probably synthesized in the mitochondria and chloroplasts of leaves and/or roots and then transferred to flowers, fruits, and meristems in plants (Arnao and Hernández-Ruiz, 2013; Tan et al., 2013). Melatonin is involved in various biological processes in plants, including circadian rhythm and photo-response regulation (Hu et al., 2016), leaf senescence (Dhindsa et al., 1981), seed germination, and root growth (Zhang et al., 2012). Meanwhile, the regulation of gene expression and crosstalk of melatonin with other phytohormones have been characterized recently, including auxin (Wang et al., 2016b; Wen et al., 2016; Arnao and Hernández-Ruiz, 2017; Arnao and Hernández-Ruiz, 2018), cytokinin (Zhang et al., 2017a), gibberellins (Zhang et al., 2014a; Zhang et al., 2014b), abscisic acid (Zhang et al., 2014a; Li et al., 2015; Fu et al., 2017), ethylene (Gao et al., 2016; Hu et al., 2016; Aghdam and Fard, 2017), jasmonic acid (Zhu and Lee, 2015), and salicylic acid (Lee et al., 2014; Shi et al., 2015a; Shi et al., 2015b; Qian et al., 2015).

A large number of experiments by using exogenous melatonin treatments indicated that melatonin plays prominent roles in abiotic stress or heavy metal tolerance in crops, vegetables, and fruits, such as the high temperature stress tolerance of cucumber seedlings (Xu et al., 2010) and tomato (Qi et al., 2018); cold stress tolerance of tomato (Ding et al., 2017) and tea plant (Li et al., 2018); salt stress tolerance of rice (Liang et al., 2015) and watermelon (Li et al., 2017); cadmium tolerance of tomato (Hasan et al., 2015) and wheat seedling (Ni et al., 2018); vanadium stress tolerance of watermelon seedlings (Nawaz et al., 2018). Besides these applications of exogenous melatonin, engineered melatonin-enriched plants generated by transgenic approach also display good properties. The transgenic *Arabidopsis* ectopically expressing MzASMT, MzSNAT5, or TaCOMT elevated the melatonin level and enhanced drought tolerance (Zuo et al., 2014; Wang et al., 2017; Yang et al., 2019); Overexpression of rice serotonin N-acetyltransferase 1 and human serotonin N-acetyltransferase in transgenic rice plants conferred resistance to cadmium and cold stress (Kang et al., 2010; Lee and Back, 2017b); *Arabidopsis* plants overexpressing alfalfa SNAT exhibited more tolerance than wild-type plants under cadmium conditions (Gu et al., 2017). Overexpression of ovine AANAT and HIOMT genes in switch grass led to improved growth performance and salt tolerance (Huang et al., 2017). Endogenous melatonin manipulation by overexpression of ASMT enhanced thermotolerance in tomato plants (Xu et al., 2016). By contrast, promotion of the post-harvest preservation of fruits and vegetables by transgenic approaches has not been studied as widely as improvement of abiotic stress resistance. However, abiotic stress tolerance resistance by transgenic approaches may give us some hints for post-harvest preservation of fruits and vegetables.

Melatonin, as a potent free radical scavenger and antioxidant (Reiter et al., 1999), protects membrane lipids and proteins against free radical damage (Tan et al., 2002). The antioxidant capacity of melatonin has been reported in some fruits and vegetables, including peaches (Gao et al., 2016; Cao et al., 2018), cassava (Ma et al., 2016), bananas (Hu et al., 2017), and cucumber (Li et al., 2016a; Xin et al., 2017), suggesting that melatonin plays important roles in post-harvest preservation of fruits and vegetables. In this review, we will focus on the emerging roles and potential application of melatonin in the post-harvest preservation of fruits and vegetables.

## Contents Of Endogenous Melatonin

### Melatonin In Post-Harvest Fruits

**Table 1** shows the analytical methods and contents of melatonin in various fruits, including apple, banana, cherry, olive, grape, cranberry, kiwi, mulberry, pineapple, pomegranate, and strawberry. The melatonin content in Merlot is 100,000–150,000 ng/g, which is the highest content among all fruits investigated. By contrast, melatonin contents in the black olive, *Prunus avium* cv. Van, *Prunus avium* cv. *Pico Limón Negro*, and *Vitis vinifera* cv. Cabernet Franc are as low as 0.1 ng/g, which are the lowest contents among all fruits listed in **Table 1**. Interestingly, Burkhardt et al. (2001) and Kirakosyan et al. (2009) measured, independently, melatonin contents in the same *Prunus cerasus* cultivars (Balaton and Montmorency), and found very different melatonin contents; its content in Balaton (1.07–2.03 ng/g) is lower than that in Montmorency (13.51–15.43 ng/g) in the Burkhardt et al. (2001) results, whereas the melatonin content in Balaton (12.3 ng/g) is higher than that in Montmorency (2.9 ng/g) in the Kirakosyan et al. (2009) results (**Table 1**). Although we do not know the exact reason for this opposite pattern of melatonin contents in these two cultivars, different measurement methods (HPLC-ECD or HPLC-EMS) used, fruit status (fresh or dry fruits), and maturation stages of fruit are likely causes of different melatonin contents. Melatonin contents are very low in other cherry cultivars, which is below 0.05 ng/g in most cases (GonzálezGómez et al., 2009). Notably, melatonin contents in the three cranberry species are extremely high, ranging from 2,500 to 9,600 ng/g (Brown et al., 2012). Most of the grape cultivars, except Merlot, Malbec, and Sangiovese, contain less than 1 ng/g melatonin, and most of the strawberry cultivars have more than 5 ng/g melatonin. These findings suggest that melatonin contents are highly variable among different species, cultivars, and organs, and are also affected by developmental and maturation stages as well as detection methods.

**Table 1:** Contents of endogenous melatonin in different post-harvest fruits.

Common name	Scientific name	Analytical method	Harvesting place/time/plant developmental stage	Melatonin content (ng/g)	References
Apple	*Malus domestica*	HPLC-FD	–	0.04 FW	Hattori et al., 1995
	*Malus pumila*	GC-MS	Egypt	0.16 FW	Badria, 2002
	Not specified	GC/MS	–	0.05 WW	Simopoulos et al., 2005
Banana	*Musa ensete*	GC-MS	Germany; Spring and Summer 1993	0.47 FW	Dubbels et al., 1995
	*Musa ensete*	GC-MS	Egypt	0.66 FW	Badria, 2002
Black olive	Not specified	LC-MS/MS	Turkey	0.01 DW	Kocadagli et al., 2014
Cherry	*Prunus cerasus* cv. Balaton	HPLC-ECD	United States; 17 July; United States; 26 July; United States; 7 August	1.07 ± 0.35 FW; 2.18 ± 0.26 FW; 2.03 ± 0.29 FW	Burkhardt et al., 2001
	*Prunus cerasus* cv. Montmorency	HPLC-ECD	United States; 17 July; United States; 26 July; United States; 7 August	13.51 ± 1.11 FW; 15.43 ± 1.75 FW; 13.96 ± 1.31 FW	Burkhardt et al., 2001
	*Prunus cerasus* cv. Balaton	HPLC-EMS	The Cherry Marketing Institute	12.3 ± 2 DW	Kirakosyan et al., 2009
	*Prunus cerasus* cv. Montmorency	HPLC-EMS	The Cherry Marketing Institute	2.9 ± 0.6 DW	Kirakosyan et al., 2009
	*Prunus avium* cv. Burlat	HPLC-MS	Spain; around mid-May	0.22 FW	GonzálezGómez et al., 2009
	*Prunus avium* cv. Navalinda	HPLC-MS	Spain; 6 days after Burlat	0.03 FW	GonzálezGómez et al., 2009
	*Prunus avium* cv. Van	HPLC-MS	Spain; 18 days after Burlat	0.01 FW	GonzálezGómez et al., 2009
	*Prunus avium* cv. Pico Limón Negro	HPLC-MS	Spain; 31 days after Burlat	0.01 FW	GonzálezGómez et al., 2009
	*Prunus avium* cv. Sweetheart	HPLC-MS	Spain; 33 days after Burlat	0.06 FW	GonzálezGómez et al., 2009
	*Prunus avium* cv. Pico Negro	HPLC-MS	Spain; 37 days after Burlat	0.12 FW	GonzálezGómez et al., 2009
	*Prunus avium* cv. Pico Colorado	HPLC-MS	Spain; 44 days after Burlat	0.05 FW	GonzálezGómez et al., 2009
	*Prunus avium* cv. Hongdeng/Rainier	SPE HPLC	IFP/BAAFS; 10-year-old Hongdeng, 12-year-old Rainier trees	10 – 20 FW	Zhao et al., 2013
Cranberry	*Vaccinium oxycoccos*	UPLC-MS	Haida Gwaii; September 2010	40,000 DW	Brown et al., 2012
	*Vaccinium vitis-idaea*	UPLC-MS	Haida Gwaii; September 2010	25,000 DW	Brown et al., 2012
	*Vaccinium macrocarpon*	UPLC-MS	Haida Gwaii; September 2010	96,000 DW	Brown et al., 2012
Grape	*Vitis vinifera* cv. Nebbiolo	HPLC-ELISA	Italy	0.97	Iriti et al., 2006
	*Vitis vinifera* cv. Croatina	HPLC-ELISA	Italy	0.87	Iriti et al., 2006
	*Vitis vinifera* cv. Barbera	HPLC-ELISA	Italy	0.63	Iriti et al., 2006
	*Vitis vinifera* cv. Cabernet Sauvignon	HPLC-ELISA	Italy	0.42	Iriti et al., 2006
	*Vitis vinifera* cv. Cabernet Franc	HPLC-ELISA	Italy	0.01	Iriti et al., 2006
	*Vitis vinifera* cv. Marzemino	HPLC-ELISA	Italy	0.03	Iriti et al., 2006
	*Vitis vinifera* cv. Sangiovese	HPLC-ELISA	Italy	0.33	Iriti et al., 2006
	*Vitis vinifera* cv. Merlot	HPLC-ELISA	Italy	0.26	Iriti et al., 2006
	*Vitis vinifera* cv. Cabernet Sauvignon/Malbec	CEC	Argentina; April 2009	0.6 – 1.2	Stege et al., 2010
	*Vitis vinifera* cv. Merlot	UPLC-(ToF)MS	British, 21–30 August 2008	100,000 – 150,000 FW	Murch et al., 2010
	*Vitis vinifera* cv. Malbec	HPLC-ESI-MS/MS	Gualtallary; 11-year-old plants	8.9 – 158.9	Boccalandro et al., 2011
	*Vitis vinifera* cv. Merlot	UPLC-MS/MS	Conegliano; 2009	3.9 – 9.3	Vitalini et al., 2011
	*Vitis vinifera* cv. Sangiovese	MEPS-HPLC-F	Italy	1.5 FW	Mercolini et al., 2012
	*Vitis vinifera*	MEPS-HPLC-F	Italy	1.2 FW	Mercolini et al., 2012
Kiwi	*Actinidia chinensis*	HPLC-FD	–	0.02 FW	Hattori et al., 1995
Mulberry	*Morus nigra* cv. ‘Hongguo2’ *Morus alba* cv. ‘Baiyuwang’	HPLC-ESI-MS/MS	China; 15, 21 and 28 April; 5, 12, 20, and 25 May	0.58 – 1.41 FW	Wang et al., 2016a
Pineapple	*Ananas comosus*	HPLC-FD	–	0.04 FW	Hattori et al., 1995
	*Ananas comosus*	GC-MS	Egypt	0.28 FW	Badria, 2002
	Not specified	GC/MS	–	0.04 WW	Simopoulos et al., 2005
Pomegranate	*Punica granatum*	GC-MS	Egypt	0.17 FW	Badria, 2002
Strawberry	*Fragaria magna*	HPLC-FD	–	0.01 FW	Hattori et al., 1995
	*Fragaria magna*	GC-MS	Egypt	0.14 FW	Badria, 2002
	*Fragaria ananassa* cv. Camarosa	LC-MS; LC-FD	Spain; March 2009 and April 2010	5.58 ± 0.01 FW	Stürtz et al., 2011
	*Fragaria ananassa* cv. Candonga	LC-MS; LC-FD	Spain; March 2009 and April 2010	5.5 ± 0.6 FW	Stürtz et al., 2011
	*Fragaria ananassa* cv. Festival	LC-MS; LC-FD	Spain; March 2009 and April 2010	11.26 ± 0.13 FW	Stürtz et al., 2011
	*Fragaria ananassa* cv. Primoris	LC-MS; LC-FD	Spain; March 2009 and April 2010	8.5 ± 0.6FW	Stürtz et al., 2011
	Not specified	GC/MS	–	0.01 WW	Simopoulos et al., 2005

FW, fresh weight; DW, dry weight; WW, wet weight.

### Melatonin In Post-Harvest Vegetables

Similar to post-harvest fruits, endogenous melatonin contents in post-harvest vegetables vary greatly depending on different species (**Table 2**). Most vegetables contain less than 10 ng melatonin per gram vegetable. For examples, the amount of endogenous melatonin is 0.299 ng/g in onion, 0.309 ng/g in cabbage, 0.5 ng/g in carrot, 0.59 ng/g cucumber, and 0.82 ng/g in Cauliflower (Badria, 2002). The highest melatonin content in vegetables was found in mushroom *Lactarius deliciosus* (12,900 ng/g). The second highest melatonin levels were observed in beans, such as lentil (1,089.8 ng/g) and kidney bean (529 ng/g) (Aguilera et al., 2015; Aguilera et al., 2016). The melatonin content in pepper is 31.0–93.4 ng/g as measured by UHPLC-QqQ-MS/MS (Riga et al., 2014), differing from those in hybrid cultivars Sena (9.1–42.1 ng/g) and Mert (7.3–31.2 ng/g) as determined by HPLC-FD (Korkmaz et al., 2014), which may be due to the differences in maturity stage, cultivars, and detection methods. Moreover, expression levels of melatonin synthesis-related genes at midnight was much higher than that at noon (Wei et al., 2016), which indicates that melatonin is more abundantly synthesized at night than at daytime. In general, endogenous melatonin contents in vegetables are relatively higher than those in fruits (**Tables 1** and **2**). However, the melatonin levels in many aromatic plants are much higher than those in usual fruits and vegetables (Chen et al., 2003; Arnao and Hernández-Ruiz, 2017). Considering the high variability of melatonin contents in different species, cultivars, and organs, which are affected by growth and maturation stages and circadian changes, it is necessary to evaluate melatonin contents comprehensively and accurately when we perform the melatonin content measurements.

**Table 2:** Contents of endogenous melatonin in different post-harvest vegetables.

Common name	Scientific name	Tissue	Analytical method	Harvesting place/time/plant developmental stage	Melatonin content (ng/g)	References
Anise	*Pimpinela anisum*	Seeds	HPLC/MS	–	7 DW	Manchester et al., 2000
Asparagus	*Asparagus officinalis*	Stems	HPLC-FD	–	0.01 FW	Hattori et al., 1995
	*Asparagus officinalis*	Stems	RIA	–	9.5 FW	Hattori et al., 1995
Basidiomycota	*Armillaria mellea*	Pileus	HPLC	Poland; Autumn 2008	<10 DW	Muszynska and Sulkowska-Ziaja, 2012
	*Boletus badius*	Pileus	HPLC	Poland; Autumn 2008	<10 DW	Muszynska and Sulkowska-Ziaja, 2012
	*Boletus edulis*	Pileus	HPLC	Poland; Autumn 2008	6,800 ± 60 DW	Muszynska and Sulkowska-Ziaja, 2012
	*Cantharellus cibarius*	Pileus	HPLC	Poland; Autumn 2008	1,400 ± 110 DW	Muszynska and Sulkowska-Ziaja, 2012
	*Lactarius deliciosus*	Pileus	HPLC	Poland; Autumn 2008	12,900 ± 770 DW	Muszynska and Sulkowska-Ziaja, 2012
	*Pleurotus ostreatus*	Pileus	HPLC	Poland; Autumn 2008	<10 DW	Muszynska and Sulkowska-Ziaja, 2012
	*Agaricus bisporus*	Pileus	RP-HPLC	–	4,300 – 6,400 DW	Muszynska et al., 2016
Beetroot	*Beta vulgaris*	Roots	GC/MS	Germany	0.002	Dubbels et al., 1995
Black mustard	*Brassica nigra*	Seeds	HPLC/MS	–	129 DW	Manchester et al., 2000
Cabbage	*Brassica oleracea*	Leaves	HPLC-FD	–	0.11 FW	Hattori et al., 1995
	*Brassica oleraceae* cv. Capitata	Leaves	GC-MS	Egypt	0.31 FW	Badria, 2002
Chinese cabbage	*Raphamus sativas*	Leaves	HPLC-FD	–	0.11 FW	Hattori et al., 1995
Cardamom	*Elettaria cardamomum*	Seeds	HPLC/MS	–	15 DW	Manchester et al., 2000
Carrot	*Daucus carota*	Roots	GC-MS	Egypt	0.50 FW	Badria, 2002
	*Daucus carota*	Roots	HPLC-FD	–	0.06 FW	Hattori et al., 1995
	Not specified	Roots	GC/MS	–	0.06 WW	Simopoulos et al., 2005
Cauliflower	*Brassica oleraceae* cv. botrytis	Flowers	GC-MS	Egypt	0.82 FW	Badria, 2002
Celery	*Apium gravolens*	Seeds	HPLC/MS	–	7 DW	Manchester et al., 2000
Coriander	*Coriandrum sativum*	Seeds	HPLC/MS	–	7DW	Manchester et al., 2000
Cucumber	*Cucumis sativus*	Fruits	GC-MS	Egypt	0.59 FW	Badria, 2002
	*Cucumis sativus*	Fruits	HPLC-FD	–	0.03 FW	Hattori et al., 1995
	*Cucumis sativus*	Fruits	GC-MS	Germany	0.10 FW	Dubbels et al., 1995
	Not specified	Fruits	GC/MS	–	0.03 WW	Simopoulos et al., 2005
Date palm	*Phoenix dactylifera*	Fruits	HPLC	Spain	0.01 – 0.17FW	Verde et al., 2018
Fennel	*Foeniculum vulgare*	Seeds	HPLC/MS	–	28 DW	Manchester et al., 2000
Fenugreek	*Brassica nigra*	Seeds	HPLC/MS	–	43 DW	Manchester et al., 2000
Garlic	*Allium sativum*	Bulbs	GC-MS	Egypt	0.59 FW	Badria, 2002
Ginger	*Zingiber officinale*	Roots	HPLC-FD	–	0.58 FW	Hattori et al., 1995
	*Zingiber officinale*	Roots	GC-MS	Egypt	1.42 FW	Badria, 2002
Kidney bean	*Phaseolus vulgaris* cv. Pinta	Cotyledon	ELISA	Spain	529.1 ± 27.5 DW	Aguilera et al., 2016
	*Phaseolus vulgaris* cv. Pinta	Cotyledon	HPLC-MS/MS	Spain	529 DW	Aguilera et al., 2015
Lentil	*Lens culinaris* cv. Salmantina	Seeds	HPLC-MS/MS	Spain	1,089.8 DW	Aguilera et al., 2015
	Not specified	Seeds	RIA	–	0.92 ± 0.06 DW	
Milk thistle	*Silybum marianum*	Seeds	HPLC/MS	–	2 DW	Manchester et al., 2000
						
Onion	*Allium fistulosum*	Bulbs	HPLC-FD	–	0.09 FW	Hattori et al., 1995
	*Allium cepa*	Bulbs	HPLC-FD	–	0.03 FW	Hattori et al., 1995
	*Allium cepa*	Bulbs	GC-MS	Egypt	0.30 FW	Badria, 2002
	Not specified	Bulbs	GC/MS	–	0.03 WW	Simopoulos et al., 2005
Pepper	*Capsicum annuum* cv. Sena	Fruits	HPLC–FD	Turkey; 6 DAF Turkey; 30 DAF Turkey; red mature stage	31.7 – 42.1 FW 9.1 FW 20.1 FW	Korkmaz et al., 2014
	*Capsicum annuum* cv. Mert	Fruits	HPLC–FD	Turkey; 6 DAF Turkey; 26 DAF Turkey; red mature stage	31.2 FW 7.3 FW 19.8 FW	Korkmaz et al., 2014
	*Capsicum annuum* cv. Barranca	Fruits	UHPLC-MS/MS	Spain	4.48 FW, 31.01 DW	Riga et al., 2014
	*Capsicum annuum* cv. Cristal	Fruits	UHPLC-MS/MS	Spain	7.72 FW	Riga et al., 2014
	*Capsicum annuum* cv. F26	Fruits	UHPLC-MS/MS	Spain	11.9 FW, 93.4 DW	Riga et al., 2014
	*Capsicum annuum* cv. Velero	Fruits	UHPLC-MS/MS	Spain	6.23 FW	Riga et al., 2014
Purslane	*Portulaca oleracea*	Leaves	GC/MS	–	19 WW	Simopoulos et al., 2005
Radish	*Bassica campestris*	Fruits	HPLC-FD	–	0.66 FW	Hattori et al., 1995
	*Raphanus sativus*	Roots	HPLC/MS	–	0.6 – 485	Chen et al., 2003
	*Raphanus sativus*	Fruits	GC-MS	Egypt	0.76 FW	Badria, 2002
Spinach	*Basella alba*	Leaves	HPLC-FD	–	0.04 FW	Hattori et al., 1995
	Not specified	Leaves	GC/MS	–	0.04 WW	Simopoulos et al., 2005
Soya bean	*Glycine max*	Seeds	RIA	–	1.89 ± 0.11 DW	
Taro	*Colocasis escutenta*	Corm	HPLC-FD	–	0.06 FW	Hattori et al., 1995
Tomato	*Lycopersicon pimpinellifolium*	Fruits	GC-MS	Germany	0.11	Dubbels et al., 1995
	*Lycopersicon esculentum Mill*. cv. Sweet 100	Fruits	GC/MS	Germany	0.51	Dubbels et al., 1995
	*Lycopersicon esculentum Mill*. cv. Rutgers California Supreme	Fruits	GC/MS	Germany	0.17	Dubbels et al., 1995
	*Solanum lycopersicum* cv. Micro-Tom	Fruits	EIA	–	1.5 – 66.6 FW	Okazaki and Ezura, 2009
	*Solanum lycopersicum* cv. Micro-Tom	Fruits	HPLC	About 34 DAF	6.58 FW	Wang et al., 2014
	*Solanum lycopersicum* cv. Micro-Tom	Fruits	HPLC	About 34 DAF	7.39 – 10.34 FW	Wang et al., 2014
	*Solanum lycopersicum*	Fruits	RIA	–	0.03	Hattori et al., 1995
	*Lycopersicon esculentum*	Fruits	HPLC-FD	–	0.03 FW	Hattori et al., 1995
	*Lycopersicon pimpinellifolium*	Fruits	LC-MS	Egypt	0.302 FW	Badria, 2002
	*Lycopersicon esculentum* cv. Borsalina	Fruits	LC-MS; LC-FD	Spain; January 2009	8.2 ± 0.6 FW	Stürtz et al., 2011
	*Lycopersicon esculentum* cv. Bond	Fruits	LC-MS; LC-FD	Spain; January 2009	23.87 ± 2.02 FW	Stürtz et al., 2011
	*Lycopersicon esculentum* cv. Catalina	Fruits	LC-MS; LC-FD	Spain; January 2009	4.1 ± 0.9 FW	Stürtz et al., 2011
	*Lycopersicon esculentum* cv. Gordal	Fruits	LC-MS; LC-FD	Spain; January 2009	17.10 ± 1.21 FW	Stürtz et al., 2011
	*Lycopersicon esculentum* cv. Lucinda	Fruits	LC-MS; LC-FD	Spain; January 2009	4.45 ± 0.05 FW	Stürtz et al., 2011
	*Lycopersicon esculentum* cv. Marbone	Fruits	LC-MS; LC-FD	Spain; January 2009 Spain; February 2010	18.13 ± 2.24 FW 114.5 ± 3.7 FW	Stürtz et al., 2011
	*Lycopersicon esculentum* cv. Myriade	Fruits	LC-MS; LC-FD	Spain; January and February 2009	8.0 ± 1.3 FW	Stürtz et al., 2011
	*Lycopersicon esculentum* cv. Pitenza	Fruits	LC-MS; LC-FD	Spain; January 2009 Spain; February 2010	14.2 ± 0.7 FW 14.0 ± 2.5 FW	Stürtz et al., 2011
	*Lycopersicon esculentum* cv. Santonio	Fruits	LC-MS; LC-FD	Spain; January and February 2009	7.73 ± 1.22 FW	Stürtz et al., 2011
	*Lycopersicon esculentum* cv. Platero	Fruits	LC-MS; LC-FD	Spain; February 2010	13.6 ± 2.5 FW	Stürtz et al., 2011
	*Lycopersicon esculentum* cv. RAF	Fruits	LC-MS; LC-FD	Spain; February 2010	50.1 ± 6.7 FW	Stürtz et al., 2011
	*Solanum lycopersicum* cv. Ciliegia	Fruits	UHPLC-MS/MS	Spain	0.64 FW 7.47 DW	Riga et al., 2014
	*Solanum lycopersicum* cv. Optima	Fruits	UHPLC-MS/MS	Spain	14.77 FW 249.98 DW	Riga et al., 2014
	Not specified	Fruits	LC-MS/MS	Germany	0.03 ± 0.01 DW	Kocadagli et al., 2014
Turnip	*Brassica rapa*	Roots	GC-MS	Egypt	0.50 FW	Badria, 2002
Vetch	*Vicia sativa*	Seeds	RIA	–	1.91 ± 0.11 DW	
White mustard	*Brassica hirta*	Seeds	HPLC/MS	–	189 DW	Manchester et al., 2000

FW, fresh weight; DW, dry weight; WW, wet weight; DAF, day after flowering.

## Application Of Exogenous Melatonin

Because the amount of endogenous melatonin is generally low in fruits and vegetables (**Tables 1** and **2**), its low content may not be enough to improve the post-harvest preservation. Currently, there is no direct research evidence showing that the shelf-life of post-harvest fruits and vegetables can be prolonged by changing endogenous melatonin contents. However, exogenous melatonin is utilized for improving the post-harvest preservation, aside from traditional means such as storage at low-temperatures and under dark conditions.

### Application In Post-Harvest Fruits

As summarized in **Table 3**, the application of exogenous melatonin in several fruits can improve their post-harvest preservation. The gray mold caused by *Botrytis cinerea* is one of the main diseases in apple during post-harvest storage, which significantly shortens the shelf-life. Application of 200 µM exogenous melatonin for 72 h inhibits gray mold (Cao et al., 2017), indicating that melatonin can prolong the shelf-life of fruits by preventing fungal infection. In addition, the application of exogenous melatonin can reduce apple juice browning, enhance anti-microorganism activity, and prolong the shelf-life of apple juice (Zhang et al., 2018a). Hu et al. (2017) soaked bananas in different concentrations (from 50 to 500 µM) of melatonin solution and found that the storage time of bananas was increased in a concentration-dependent manner. Notably, ‘nan Tian Huang’ and ‘Bao Dao’ bananas can be stored for 16 days when treated with 500 µM melatonin, whereas untreated bananas can be stored for only 4–5 days (Hu et al., 2017). After treatment with 100 µM melatonin solution for 10 min, the senescence of peach fruit was delayed, and chilling-induced flesh browning was well controlled, via the melatonin-mediated regulation on reactive oxygen species, membrane fatty acid contents, and phenolic metabolism (Gao et al., 2016; Gao et al., 2018). Moreover, peaches treated with 100 µM melatonin solution for 2 h could be stored for 28 days with higher levels of total soluble solids and extractable juice rate than the non-treated peaches (Cao et al., 2016). Atomic-force-microscopy assay revealed that the polysaccharide widths in the soluble fractions of melatonin-treated peach fruits were distributed in a shorter range compared with those in the non-treated peach fruits (Cao et al., 2018). These results show compelling evidence for a protective role of exogenous melatonin in chilling stress tolerance in post-harvest fruits, suggesting that combined applications of exogenous melatonin and low temperatures might be an effective approach for post-harvest preservation. In addition, soaking post-harvest strawberry in 100 µM melatonin solution for 2 h changes the content of antioxidant enzymes related to fruit decay, such as superoxide dismutase (SOD), ascorbate peroxidase (APX), and catalase (CAT), which results in decreased decay and senescence of strawberries (Aghdam and Fard, 2017; Liu et al., 2018). Exogenous melatonin inhibits pericarp browning in lychee (*Litchi chinensis*), delays the discoloration during storage, reduces cell membrane leakage, and inhibits the production of superoxide anion (O_2_^-^), hydrogen peroxide (H_2_O_2_), and malondialdehyde (Zhang et al., 2018b). Soaking pear fruit in 100 µM melatonin solution for 12 h can effectively prolong shelf-life and prevent physiological disorders such as water immersion and nuclear browning (Zhai et al., 2018). These findings suggest that melatonin is able to significantly promote the post-harvest preservation of fruits and should be a potential target for improving post-harvest preservation of fruits in the future. However, the time, dosage, and method of melatonin treatment should be optimized according to the adverse conditions for different post-harvest fruits.

**Table 3:** Effects of exogenous melatonin on the preservation and quality of post-harvest fruits.

Common name	Scientific name	Melatonin treatment concentration (µM)	Optimum concentration (µM)	Treatment time	Treatment method	Effect	References
Apple	*Malus domestica* cv. Fuji	100; 200; 300; 400	200	6; 12; 24; 48; 72; 96; 120 h	Immersed	Gray mold ↓	Cao et al., 2017
Banana	*Musa acuminata* NTH, BD, FJ, HD	0; 50; 200; 500	200; 500	2 h	Soaked	Post-harvest banana ripening ↓	Hu et al., 2017
Peach	*Prunus persica* cv. Shahong, Qinmi	0; 100	100	10 min	Immersed	Senescence ↓; quality of peach fruit ↑	Gao et al., 2016
	*Prunus persica* Batsch cv. Chuanzhongdao	0; 100	100	10 min	Immersed	Chilling induced flesh browning ↓	Gao et al., 2018
	*Prunus persica* cv. Batsch, Hujing	0; 50; 100; 200	100	120 min	Immersed	Chilling injury ↓; extractable juice rate and total soluble solids ↑; polyamine, GABA and proline ↑	Cao et al., 2016
	*Prunus persica* cv. Batsch, Hujing	0; 100	100	2 h	Immersed	Chilling injury ↓; early stage H_2_O_2_ ↑; expression of antioxidant response genes ↑	Cao et al., 2018
Strawberry	*Fragaria × anannasa* cv. Selva	0; 1;10; 100; 1,000	100	2 h	Immersed	H_2_O_2_ accumulation ↑; SOD activity ↑; CAT and APX activities ↓; decay ↓	Aghdam and Fard, 2017
	*Fragaria × anannasa* cv. Hongyan	0; 1; 10; 100; 1,000	100; 1,000	2 h	Immersed	Senescence ↓	Liu et al., 2018
Lychee	*Litchi chinensis* cv. Sonn	50; 100; 200; 400; 800	400	5 min	Immersed	Pericarp browning ↓; discoloration during storage ↓; membrane relative leakage rate ↓; O_2_^-^, H_2_O_2_ and MDA ↓	Zhang et al., 2018b
Pear	*Pyrus communis*	1; 100	100	12 h	Immersed	Mature senescence ↓; shelf-lives ↑; water soaking ↓; core browning ↓	Zhai et al., 2018

↓ indicated decrease; ↑ indicated increase.

GABA, gamma-aminobutyric acid; H_2_O_2_, hydrogen peroxide; SOD, superoxide dismutase; CAT, catalase; APX, ascorbate peroxidase; O_2_, superoxide radical; MDA, malondialdehyde.

### Application In Post-Harvest Vegetables

Vegetables are more perishable foods than fruits, and their storage period is relatively short, usually 2–3 days (Mythili and Sathiavelu, 2010). So far, only a few studies have been conducted to evaluate effects of exogenous melatonin application on post-harvest preservation of vegetables (**Table 4**). Soaking cucumber in 100 µM melatonin solution for 2 h can keep better quality, minimize oxidative damage, delay senescence, and extend shelf life (Xin et al., 2017). Exogenous melatonin can significantly reduce H_2_O_2_ content in cassava root, resulting in delayed p*ost-harvest* physiological deterioration (PPD) symptoms caused by damage during harvest and treatment, ultimately prolonging the preservation period of vegetables (Hu et al., 2016; Ma et al., 2016). Zhu et al. (2018) soaked broccoli in 100 µM melatonin solution for 5 min and found that the yellowing index did not differ between the treatment group on the seventh day and the control group on the fifth day, indicating that melatonin treatment can extend the storage life of broccoli from 5 to 7 days. Fresh potato tuber slices pretreated with melatonin showed reduced lesion sizes of tuber slices infected by *Phytophthora infestans*, inferring that the potato late blight can be significantly attenuated by exogenous melatonin treatment (Zhang et al., 2017b). Besides the studies related to shelf-life prolongation of post-harvest vegetables and fruits, whether melatonin can promote the quality of post-harvest vegetables and fruits is remain largely unknown. A previous study showed that 50 µM melatonin treatment significantly increases lycopene contents in post-harvest tomato fruits by increasing the expression levels of phytoene synthase1 (PSY1) and carotenoid isomerase (CRTISO) that are crucial for fruit color development (Sun et al., 2015). Moreover, the total anthocyanin contents in melatonin-treated tomato fruits increased by 52%, 48%, and 50% at 5, 8, and 13 days after melatonin treatment, respectively, and eight proteins that are related to anthocyanin accumulation were increased upon melatonin application (Sun et al., 2016). Arnao and Hernandez-Ruiz (2009) found that melatonin has protective effects against chlorophyll degradation in barley. However, melatonin application studies have not been conducted on leafy vegetables. Hence, more experiments are needed to evaluate whether exogenous melatonin can be used to extend the storage life of leafy vegetables.

**Table 4:** Effects of exogenous melatonin on the preservation and quality of post-harvest vegetables.

Common name	Scientific name	Melatonin treatment concentration (µM)	Optimum concentration (µM)	Treatment time	Treatment method	Effect	References
Cucumber	*Cucumis sativus* cv. Jinyan No. 4	0; 50; 100; 500	500	2 h	Immersed	Decrease of chlorophyll, vitamin C, the content of titration-acid and soluble protein ↓	Xin et al., 2017
Cassava	*Manihot esculenta* cv. SC124	100	100	2 h	Soaked	PPD ↓; H_2_O_2_ content ↓; activities of CAT and POD during the PPD process ↑	Hu et al., 2016
	*Manihot esculenta* cv. Crantz	500	500	2 h	Incubated	SOD and CAT activities during PPD progression ↑	Ma et al., 2016
Broccoli	*Brassica oleracea* cv. Italica Planch	0; 100	100	5 min	Immersed	Storage life ↑	Zhu et al., 2018
Tomato	*Solanum lycopersicum* cv. Bmei	50	50	2 h	Immersed	Fruit ripening ↑; anthocyanin accumulation↑	Sun et al., 2016
	*Solanum lycopersicum* cv. Bmei	0; 1; 50; 100; 500	50	2 h	Immersed	Lycopene levels ↑; the expression level of PSY1 and CRTISO ↑; fruit softening ↑; ethylene production ↑; water-soluble pectin ↑; protopectin ↓	Sun et al., 2015
Potato	*Solanum tuberosum*	0; 1,000; 3,000; 6,000; 8,000; 10,000	10,000	12 h	Sprayed	Potato late blight ↓	Zhang et al., 2017a

↓ indicated decrease; ↑ indicated increase.

PPD, post-harvest physiological deterioration; H_2_O_2_, hydrogen peroxide; CAT, catalase; POD: peroxidase; SOD, superoxide dismutase; PSY1, phytoene synthase1; CRTISO, carotenoid isomerase.

In summary, exogenous melatonin can be used to improve the post-harvest preservation of fruits and vegetables. However, melatonin concentration and treatment method and time should be carefully considered when exogenous melatonin is used to improve post-harvest preservation. The most important factor is the melatonin concentration, which should be optimized to obtain the best effect. Treatment of 1,000 µM melatonin leads to spoilage and deterioration of post-harvest strawberry fruits (Aghdam and Fard, 2017), indicating that high melatonin concentration can cause a negative effect. Clearly, more studies are needed to evaluate the effects of exogenous melatonin on the post-harvest preservation of other fruits and vegetables. In addition, it would be of great interest to further determine whether exogenous melatonin can be used to preserve nutritional values of fruits and vegetables as well as their shelf-life. Because an excess amount of melatonin is harmful to post-harvest fruits (Aghdam and Fard, 2017), it also would be interesting to examine both positive and negative effects of melatonin on plant growth, development, and post-harvest storage.

## Mechanisms Of Exogenous Melatonin Functions In Post-Harvest Fruits And Vegetables

**Figure 1:**
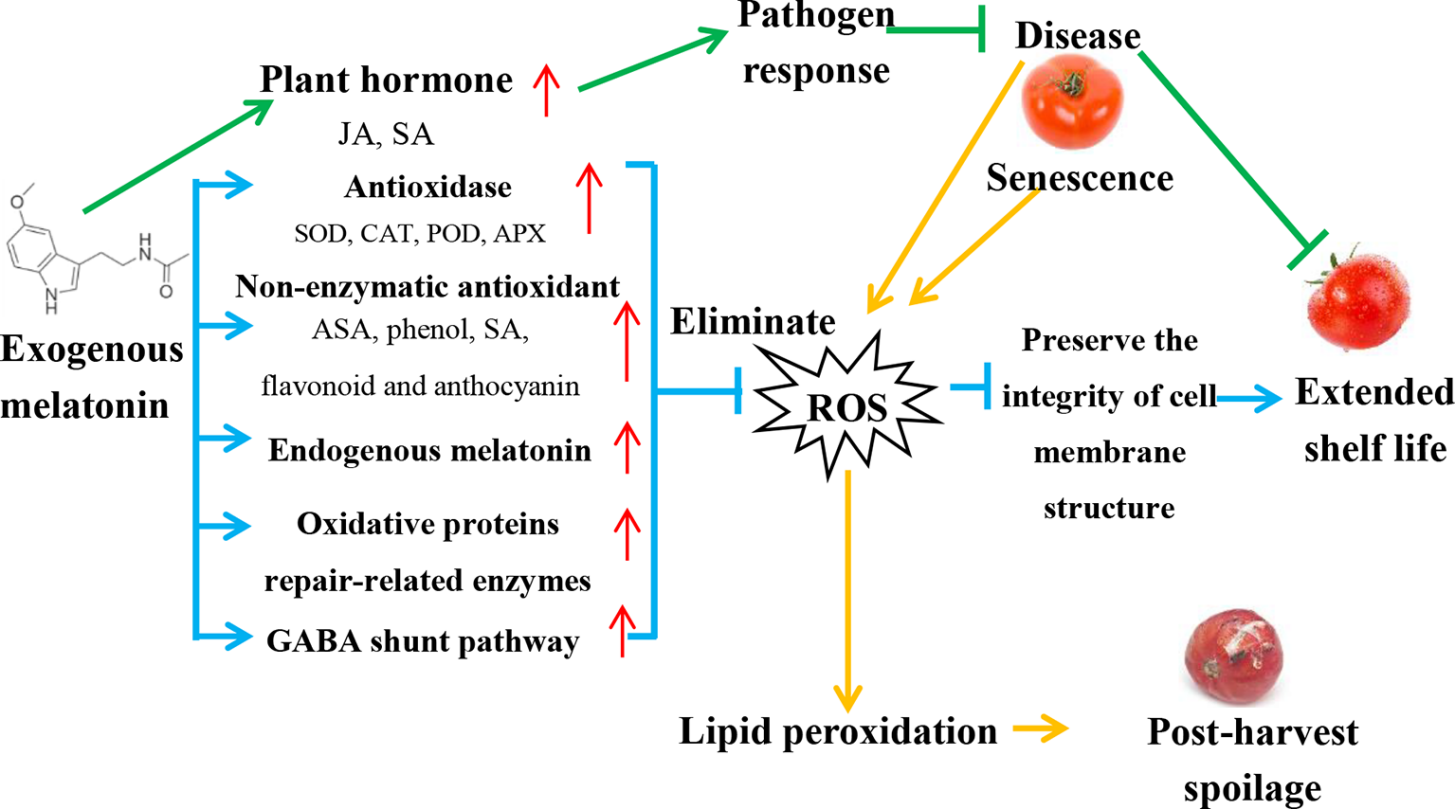
Model of exogenous melatonin-mediated post-harvest preservation mechanism in fruits and vegetables. (1) Blue lines and arrows indicate ROS elimination pathway. Melatonin acts psrimarily as a powerful free radical scavenger by increasing the content of antioxidant enzymes, non-enzymatic antioxidants, and the enzymes related to oxidative protein repair, removing excess active oxygen from post-harvest fruits and vegetables, and promoting GABA shunt pathway. Subsequently, the content of hydroxyl radicals and hydrogen peroxide decreases, the degree of membrane lipid peroxidation is reduced, thus protecting cells from oxidative damage and prolonging the shelf-life. (2) Green lines and arrows indicate pathogen response dependent pathway. Exogenous melatonin increases the levels of JA and SA, triggers plant pathogen responses, increases pathogen resistance, and extends the shelf life. (3) Orange lines and arrows indicate post-harvest decay of fruits and vegetables. Diseases or senescence of post-harvest fruits and vegetables produce lots of ROS, lead to lipid peroxidation, and cause post-harvest decay. Red arrows indicate increased levels of each component.

The senescence of fruits and vegetables is accompanied by the loss of cell membrane integrity and function, which is manifested by an increase in membrane leakage (Dumas et al., 2003). This structural/functional membrane dysfunction is caused by the excessive production of reactive oxygen species (ROS), including O2^-^, H_2_O_2_, hydroxyl radical (OH^-^), and singlet oxygen (^1^O2), which are potent compounds destroying biological macromolecules and affecting the metabolism of post-harvest fruits and vegetables. During storage, the ROS in fruits increases continuously and induces lipid peroxidation (Li et al., 2016b). Lipid peroxidation can catalyze the oxygenation of unsaturated fatty acids through ROS and lipid oxidases (such as LOX) to form volatile substances such as hydrogen peroxide derivatives (Shewfelt and del Rosario, 2000). Subsequently, these substances are decomposed to produce oxidative free radicals, triggering a chain reaction of lipid peroxidation and eventually causing the deterioration of fruits and vegetables. **Figure 1** shows a model of exogenous melatonin-mediated post-harvest preservation mechanism in fruits and vegetables, which is described in detail below.

### Exogenous Melatonin Increases Antioxidant Enzymes For Scavenging ROS

The enzymatic antioxidant system is a primary way to control the production of ROS, which regulates the degree of lipid peroxidation. SOD, CAT, and peroxidase (POD) are the key antioxidant enzymes for scavenging ROS. Cao et al. (2017) found that exogenous melatonin treatment can induce apple disease resistance by continuously increasing the activity of POD, SOD, and CAT. Melatonin upregulates the antioxidant enzyme activity of peach fruits and reduces the levels of O_2_^-^ and H_2_O_2_ in different peach varieties, thereby maintaining the metabolic balance of ROS, reducing lipid peroxidation, and delaying senescence (Gao et al., 2016). Although the expression of genes encoding antioxidant enzymes was proven to be up-regulated by melatonin treatment, the molecular mechanism underlying melatonin-mediated regulation of gene expression of antioxidant enzyme needs to be further determined. For instance, whether or not antioxidant response elements in the promoter of antioxidant enzyme genes are recognized by melatonin still remains unknown. Moreover, evaluation of oxidase activity, together with antioxidant enzyme activity, after melatonin treatment would provide further insights into the mechanistic role of melatonin as an antioxidant.

### Exogenous Melatonin Induces Non-Enzymatic Antioxidants

Besides removing excess ROS through an enzymatic antioxidant system to reduce lipid peroxidation, several non-enzymatic antioxidants also play important roles to alleviate ROS toxicity. Melatonin can act as a signaling molecule that upregulates downstream defense genes encoding many non-enzymatic antioxidants.

Ascorbic acid (ASA) is a typical non-enzymatic antioxidant that can directly scavenge ROS (Liu et al., 2015). Exogenous melatonin triggers the ASA-glutathione cycle in post-harvest peaches by upregulating the transcriptional expression of antioxidant-related enzymes, which results in increased ASA level and prolonged shelf-life of post-harvest fruits and vegetables (Song et al., 2016; Cao et al., 2018). Similar effects of melatonin on ASA-glutathione cycle were also observed in cucumber (Zhao et al., 2016). Salicylic acid (SA), another antioxidant, effectively reduces lipid peroxidation during low-temperature storage of cherry fruits (Wang et al., 2008). Liu et al. (2018) found that melatonin treatment significantly improved the clearance of 2,2-Di-(4-tert-octylphenyl)-1-picrylhydrazyl (DPPH) and 2,2’-azinobis-(3-ethylbenzothiazoline-6-sulfonic acid) (ABTS) during storage, which was positively correlated with the total phenol content and antioxidant capacity (Puerta-Gomez and Cisneros-Zevallos, 2011). Therefore, the improvement of DPPH and ABTS scavenging ability is probably related to the increased total phenol and flavonoid contents after melatonin treatment. Melatonin can increase the activity of glucose-6-phosphate dehydrogenase, shikimate dehydrogenase, and phenylalanine ammonia-lyase that promote the accumulation of total phenols and endogenous SA, which is beneficial to inhibit fungal decay and to prolong the shelf-life of post-harvest peach (Gao et al., 2018). Phenolic compounds can protect membrane lipids from peroxidation by preventing the occurrence and propagation of oxidative chain reactions (Pennycooke et al., 2005). In addition, the delayed increase of browning-related enzymes such as polyphenol oxidase in lychee fruits upon melatonin treatment was consistent with the increase in total phenol, flavonoid, and anthocyanin contents, suggesting that melatonin can inhibit enzymatic phenol oxidation and delay pericarp browning of lychee fruits (Zhang et al., 2018b). Moreover, exogenous melatonin treatment significantly induces the accumulation of endogenous melatonin (Aghdam et al., 2016; Liu et al., 2018; Zhang et al., 2018). These findings indicate that endogenous melatonin is not only a strong antioxidant that delays the senescence of fruits and vegetables but also a signal molecule that mediates the antioxidant capacity of non-enzymatic as well as enzymatic antioxidants.

### Exogenous Melatonin Increases Oxidative Protein Repair-Related Enzymes

Under environmental stress and senescing process, ROS damages the conformations and functions of proteins through the oxidative modification of amino acid side chains. Methionine and cysteine are sulfur-containing amino acids that are highly susceptible to damage caused by ROS. Living organisms have evolved to acquire the methionine sulfoxide reductase (Msr) system that can repair oxidized proteins (Vogt, 1995; Baraibar and Friguet, 2013). Zhang et al. (2018b) found that treatment of 400 µM melatonin markedly enhances the expression of *LcMsrA1*, *LcMsrA2*, *LcMsrB1*, and *LcMsrB2* in lychee fruits during storage and significantly extends the duration of viable protein repair mechanisms and protection from ROS. Yu et al. (2016) found that the heat resistance of ‘Jersey’ blueberry is improved by increasing the transcription level of oxidative protein repair-related genes. At present, there are relatively few studies on the enzyme repair system of oxidized proteins that are related to the post-harvest preservation of fruits and vegetables. With the advancement of omics technologies including transcriptomes, proteomes, and metabolomes, future studies would reveal novel functions of oxidative protein repair-related enzymes and the role of melatonin in the regulation of the Msr system in the post-harvest fruits and vegetables.

### Relationship Between Exogenous Melatonin and Hormones in Post-Harvest Stage

Excessive ethylene synthesis produces more ROS, causes lipid peroxidation, accelerates aging, and ultimately leads to post-harvest decay of fruits and vegetables. Zhai et al. (2018) found that ethylene levels in ‘Starkrimson’ pears are highly increased during post-harvest storage for 1–3 days, but its level could be reduced and maintained in a stable state by adding 100 µM melatonin externally. Exogenous melatonin could reduce the production of ethylene in four varieties of bananas during post-harvest ripening (Hu et al., 2017). Melatonin can effectively inhibit the expression of genes related to ethylene biosynthesis such as *Musa* 1-aminocyclopropane-1-carboxylate oxidase 1 and *Musa* 1-aminocyclopropane-1-carboxylate synthase 1, which results in delayed fruit senescence (Hu et al., 2017). These observations indicate that exogenous melatonin can inhibit ethylene biosynthesis and delay the post-harvest deterioration. By contrast, melatonin promotes the formation of ethylene by up-regulating the expression of 1-aminocyclopropane-1-carboxylic acid synthase, thereby accelerating the ripening of tomatoes (Klee and Giovannoni, 2011). Exogenous melatonin treatment slightly promotes ethylene synthesis and the subsequent timing of the climacteric peak (Sun et al., 2015). At same time, melatonin up-regulates the expression of ethylene receptor genes (ETR4 and NR), transducing elements (ERF2, EIL3, and EIL1), cell wall changes, pigments, and flavor accumulation, but down-regulates fruit senescence-related proteins and antioxidant enzyme proteins (Sun et al., 2015; Sun et al., 2016). Notably, melatonin is positively correlated with fruit ripening but is negatively correlated with climacteric fruit senescence (Zhai et al., 2018). These findings suggest that melatonin plays a unique role in maturation and senescence and balances the production of ethylene. Moreover, the effects of melatonin on the ripening and preservation of post-harvest fruits and vegetables also depend on their maturity and starch content.

The decay of post-harvest fruits and vegetables is usually accompanied with pathogen infection. Melatonin has antioxidant, immune modulatory, and anti-inflammatory effects, suggesting that it has an ability for inhibiting bacterial, viral, and parasitic infections. Exogenous melatonin can prolong the shelf-life of fruits by preventing fungal infection (Cao et al., 2017). Melatonin treatment contributed to greater resistance to fungal infection in apple trees (Yin et al., 2013) and *Lupinus albus* (Arnao and Hernández-Ruiz, 2015). Melatonin increases the expression of pathogenesis-, nitric oxide (NO)-, and salicylic acid (SA)-related genes, and leads to an increased resistance to the pathogen, indicating that melatonin may be a signaling molecule in pathogen response (Arnao and Hernández-Ruiz, 2018). Melatonin synthesis gene *SNAT* knockout mutants with reduced melatonin and SA level showed greater susceptibility to the pathogen (Lee et al., 2014; Lee et al., 2015), suggesting that melatonin and SA are likely to have a synergistic effect on fruits and vegetables after harvest. Melatonin acts upstream of the pathogen resistance signaling pathway, induces the biosynthesis of NO, ethylene, JA, and SA, then elicits disease resistance (Zhu and Lee, 2015). It appears that melatonin is involved in innate plant immunity against fungal and bacterial pathogens via an SA/JA/ethylene and NO-dependent pathway (Lee et al., 2014; Qian et al., 2015; Shi et al., 2015a; Shi et al., 2015b; Shi et al., 2016; Lee and Back, 2017). Although most of these findings in plants are not directly related to post-harvest fruits and vegetables, they suggest that crosstalk between melatonin, JA, SA, and NO is deeply related to pathogen responses during the post-harvest fruits and vegetables deterioration.

Indole acetic acid (IAA) is a plant growth hormone that can promote the expansion and vacuolation of plant cells. Biosynthesis of melatonin and IAA starts from tryptophan, indicating that melatonin and IAA compete for the same starting precursor (Hernandez-Ruiz et al., 2004; Hernandez-Ruiz et al., 2005, Hernandez-Ruiz and Arnao, 2008a; Hernandez-Ruiz and Arnao, 2008b; Arnao and Hernández-Ruiz, 2017). Experiments have shown that IAA accumulates at the third growth stage in cherries (35 days after flowering) (Wang et al., 2008), whereas melatonin level declines at the same growth stage (Zhao et al., 2013). Chen et al. (2009) found that exogenous melatonin treatment increases the level of endogenous free IAA and effectively promotes the development of seedling roots, indicating that melatonin and IAA have synergistic effects. Previous studies have shown that IAA can delay the ripening and senescence of bananas (Vendrell, 1969), pears (Frenkel and Dyck, 1973), and avocados (Tingwa and Young, 1975). Although the addition of a suitable concentration of melatonin can prolong the shelf-life of post-harvest fruits and vegetables, no direct studies have shown that IAA has similar effects. Because melatonin is a hormone secreted by pineal gland in animals, it would be of interest to further investigate whether melatonin functions as a phytohormone and interacts with other plant hormones during post-harvest process.

### Exogenous Melatonin Activates the γ-Aminobutyric Acid (GABA) Shunt Pathway

GABA is a four-carbon non-proteinogenic amino acid widely found in plants and is mainly produced by the GABA shunt of the (TCA) Tricarboxylic Acid cycle. Three enzymes, glutamine decarboxylase, GABA transaminase (GABA-T), and succinic semialdehyde dehydrogenase (SSADH) are involved in this pathway (Bown and Shelp, 1997; Bouché and Fromm, 2004). GABA shunt can inhibit the accumulation of ROS and reduce the sensitivity of *ssadh Arabidopsis* mutants to environmental stress (Bouché et al., 2003). Exogenous melatonin can increase the activity of GABA-T enzyme by providing more NADH and succinic acid for the TCA cycle and mitochondrial electron transport chain. This in turn produces more ATP for fruits and vegetables to remove excess H_2_O_2_ and ROS, thus prolonging the shelf-life of post-harvest fruits (Bouché et al., 2003; Carvajal et al., 2015; Palma et al., 2015). Aghdam and Fard (2017) has found that exogenous melatonin can improve the GABA shunt pathway, thereby enhancing the inhibition of the post-harvest decay of strawberry fruits. Palma et al. (2015) suggested that higher GABA shunt pathway and GABA-T activity can contribute to the chilling tolerance in Natura zucchini fruit during storage at 4°C. Because it is evident that the endogenous level of melatonin increases in response to diverse stresses, including cold, drought, oxygen deprivation, and high salinity (Arnao and Hernández-Ruiz, 2013), it would be interesting to further determine whether melatonin can exert its stress-tolerance functions by modulating GABA shunt pathway during the storage of fruits and vegetables under stressful conditions.

### Melatonin Acts As a Signal Molecule

In *Arabidopsis*, exogenous melatonin can induce many defense related genes, such as PR protein 1 (PR1), plant defensin 1.2 (PDF1.2), 1-aminocyclopropane-1-carboxylatesynthase 6 (ACS6), isochorismate synthase 1 (ICS1), ascorbate peroxidase 1 (APX1), vegetative storage protein 1 (VSP1), and glutathione-S-transferase 1 (GST1), and then suppress the bacterial propagation, implying melatonin as an endogenous signal molecule triggering defense responses against pathogen attack (Lee et al., 2014). Moreover, it was found that melatonin functions downstream of H_2_O_2_ and NO, and upstream of the serine/threonine-protein kinase and MAPKKK kinases, indicating that melatonin is required for H_2_O_2_- and NO- mediated defense signaling (Lee and Back, 2017). A large number of genes, involved in MAPK signaling, nucleotide metabolism, and ethylene biosynthesis, are transcriptionally reprogrammed by melatonin treatment, suggesting the possible role of melatonin as a signal molecule (Xu et al., 2017). Furthermore, exogenous melatonin treatment could promote methyl jasmonate (MeJA) accumulation and enhance the expression of proteinase inhibitor II in tomato fruits, which results in the up-regulation of JA defense signaling that is a crucial pathway in pathogen resistance (Liu et al., 2019). Other studies showed that melatonin is involved in abscisic acid and cytokinin metabolic signaling pathways regulating heat stress response in perennial ryegrass (Zhang et al., 2017a), and participates in adventitious root development by regulating auxin and nitric oxide signaling in tomato plants (Wen et al., 2016). These results clearly indicate that melatonin acts as a signal molecule in many biological processes in plants. However, the role of melatonin as a signal molecule remains largely unknown in the preservation process of post-harvest fruits and vegetables, which needs further demonstration in the future.

## Conclusions And Future Prospects

Many recent studies clearly point to the prominent roles of melatonin in the preservation of post-harvest fruits and vegetables, which primarily rely on melatonin’s activity to scavenge ROS by increasing antioxidant enzymes and non-enzymatic antioxidants. Melatonin can also up-regulate the expression of genes encoding oxidative protein repair-related enzymes to maintain redox homeostasis in fruit and vegetable cells, which indicates that endogenous melatonin functions as a strong antioxidant to remove excessive ROS. Notably, application of exogenous melatonin can activate endogenous melatonin synthesis and induce other molecular signals that lead to anti-aging effects. These findings provide a valuable scientific basis for future research aiming at extending shelf-life of fruits and vegetables. Although exogenous melatonin can be used to increase the preservation period of post-harvest fruits and vegetables, it would be of great interest to determine whether the shelf-life of fruits and vegetables can be prolonged by increasing endogenous melatonin via transgenic approach. Given that genes involved in melatonin biosynthesis have been identified in several plants, it would be worthy to identify orthologous genes in fruits and vegetables and to engineer target crops for higher melatonin content by overexpressing key genes involved in melatonin biosynthesis. Because our knowledge on the function of melatonin in the post-harvest preservation is far from sufficient, more studies are needed to determine the mechanistic role of melatonin in post-harvest storage. Moreover, the combined effects of melatonin and classical preservation technologies should also be evaluated for practical application of melatonin in post-harvest storage of fruits and vegetables.

## Author Contributions

TX and HK designed the concept. TX and YC wrote the manuscript. HK revised the manuscript. All authors read and approved the manuscript.

## Funding

This work was supported jointly by grants from the National Natural Science Foundation of China (Grant No. 31701481), the Natural Science Foundation of Jiangsu Province (No. BK20160214), the Natural Science Foundation of Jiangsu Higher Education Institutions of China (19KJA510010), the Priority Academic Program Development of Jiangsu Higher Education Institutions (No. PAPD) and Jiangsu Overseas Visiting Scholar Program for University Prominent Young & Middle-aged Teachers and Presidents.

## Conflict of Interest

The authors declare that the research was conducted in the absence of any commercial or financial relationships that could be construed as a potential conflict of interest.

## References

[B1] Adeyeye O. A. Sadiku E. R. Selvam P. Perumal A. B. Nambiar R. (2017). Post-harvest preservation of mango using tray and freeze drying methods. Soc. Sci. Electron. Publ. 10, 09.

[B2] Aghdam M. S. Fard J. R. (2017). Melatonin treatment attenuates post-harvest decay and maintains nutritional quality of strawberry fruits (*Fragaria j12 × anannasa* cv. Selva) by enhancing GABA shunt activity. Food Chem. 221, 1650–1657. 10.1016/j.foodchem.2016.10.123 27979142

[B3] Aghdam M. S. Naderi R. Malekzadeh P. Jannatizadeh A. (2016). Contribution of GABA shunt to chilling tolerance in anthurium cut flowers in response to postharvest salicylic acid treatment. Sci. Hortic-Amsterdam 205, 90–96. 10.1016/j.scienta.2016.04.020

[B4] Aguilera Y. Herrera T. Liebana R. Rebollo-Hernanz M. Sanchez-Puelles C. Martin-Cabrejas M. A. (2015). Impact of melatonin enrichment during germination of legumes on bioactive compounds and antioxidant activity. J. Agric. Food Chem. 63, 7967–7974. 10.1021/acs.jafc.5b03128 26307852

[B5] Aguilera Y. Rebollo-Hernanz M. Herrera T. Cayuelas L. T. Rodriguez-Rodriguez P. de Pablo A. L. L. (2016). Intake of bean sprouts influences melatonin and antioxidant capacity biomarker levels in rats. Food Funct. 7, 1438–1445. 10.1039/c5fo01538c 26841704

[B6] Arnao M. B. Hernández-Ruiz J. (2015). Functions of melatonin in plants: a review. J. Pineal Res. 59, 133–150. 10.1111/jpi.12253 26094813

[B7] Arnao M. B. Hernández-Ruiz J. (2017). Growth activity, rooting capacity, and tropism: three auxinic precepts fulfilled by melatonin. Acta Physiol Plant 39, 127. 10.1007/s11738-017-2428-3

[B8] Arnao M. B. Hernandez-Ruiz J. (2013). Growth conditions determine different melatonin levels in *Lupinus albus L*. J. Pineal. Res. 55, 149–155. 10.1111/jpi12055 23600673

[B9] Arnao M. B. Hernández-Ruiz J. (2013). Growth conditions influence the melatonin content of tomato plants. Food. Chem. 138, 1212–1214. 10.1016/j.foodchem.2012.10.077 23411233

[B10] Arnao M. B. Hernández-Ruiz J. (2018). Melatonin and its relationship to plant hormones. Ann Botlondon. 121, 195–207. 10.1093/aob/mcx114 PMC5808790 29069281

[B11] Arnao M. B. Hernández-Ruiz J. (2017). “Phyto-melatonin: a natural substance from plants with interesting nutraceutical properties,” in Nutraceuticals: Prospects, Sources and Role in Health and Disease. Ed. (New York: NOVA Science Publ), 123–157.

[B12] Arnao M. B. Hernandez-Ruiz J. (2009). Protective effect of melatonin against chlorophyll degradation during the senescence of barley leaves. J. Pineal. Res. 46, 58–63. 10.1111/j.1600-079X.2008.00625.x 18691358

[B13] Badria F. A. (2002). Melatonin, serotonin, and tryptamine in some Egyptian food and medicinal plants. J. Med. Food 5, 153–157. 10.1089/10966200260398189 12495587

[B14] Baraibar M. A. Friguet B. (2013). Oxidative proteome modifications target specific cellular pathways during oxidative stress, cellular senescence and aging. Exp. Gerontol. 48, 620–625. 10.1016/j.exger.2012.10.007 23127722

[B15] Barrett D. M. Lloyd B. (2012). Advanced preservation methods and nutrient retention in fruits and vegetables. J. Sci. Food Agr. 92, 7–22. 10.1002/jsfa.4718 22102258

[B16] Boccalandro H. E. Gonzalez C. V. Wunderlin D. A. Silva M. F. (2011). Melatonin levels, determined by LC-ESI-MS/MS, fluctuate during the day/night cycle in *Vitis vinifera* cv. Malbec: evidence of its antioxidant role in fruits. J. Pineal Res. 51, 226–232. 10.1111/j.1600-079X.2011.00884.x 21605162

[B17] Bouché N. Fait A. Bouchez D. Møller S. G. Fromm H. (2003). Mitochondrial succinic-semialdehyde dehydrogenase of the γ-aminobutyrate shunt is required to restrict levels of reactive oxygen intermediates in plants. P. Natl. Acad. Sci. U. S. A. 100, 6843–6848. 10.1073/pnas.1037532100 PMC164534 12740438

[B18] Bouché N. Fromm H. (2004). GABA in plants: just a metabolite? Trends Plant Sci. 9, 110–115. 10.1016/j.tplants.2004.01.006 15003233

[B19] Bown A. W. Shelp B. J. (1997). The metabolism and functions of [gamma]- aminobutyric acid. Plant Physiol. 115, 1–5. 10.1104/pp.115.1.1 12223787 PMC158453

[B20] Brown P. N. Turi C. E. Shipley P. R. Murch S. J. (2012). Comparisons of large (*Vaccinium macrocarpon* Ait.) and small (*Vaccinium oxycoccos L*., *Vaccinium vitis-idaea L*.) cranberry in British Columbia by phytochemical determination, antioxidant potential, and metabolomic profiling with chemometric analysis. Planta Med. 78, 630–640. 10.1055/s-0031-1298452 22337317

[B21] Bureau S. Chahine H. Gouble B. Reich M. Albagnac G. Audergon J. M. (2006). Fruit ripening of contrasted apricot varieties: physical, physiological and biochemical changes. Acta Hortic. 701, 511–516. 10.17660/ActaHortic.2006.701.88

[B22] Burkhardt S. Tan D. X. Manchester L. C. Hardeland R. Reiter R. J. (2001). Detection and quantification of the antioxidant melatonin in Montmorency and Balaton tart cherries (*Prunus cerasus*). J. Agr. Food Chem. 49, 4898–4902. 10.1021/jf010321+ 11600041

[B23] Cao J. J. Yu Z. C. Zhang Y. Li B. H. Liang W. X. Wang C. X. (2017). Control efficiency of exogenous melatonin against post-harvest apple grey mold and its influence on the activity of defensive enzymes. Plant Physiol. J. 53, 1753–1760. 10.13592/j.cnki.ppj.2017.0197

[B24] Cao S. F. Shao J. R. Shi L. Y. Xu L. W. Shen Z. M. Chen W. (2018). Melatonin increases chilling tolerance in post-harvest peach fruit by alleviating oxidative damage. Sci. Rep-UK. 8, 806. 10.1038/s41598-018-19363-5 PMC5770464 29339757

[B25] Cao S. F. Song C. B. Shao J. R. Bian K. Chen W. Yang Z. F. (2016). Exogenous melatonin treatment increases chilling tolerance and induces defense response in harvested peach fruit during cold storage. J. Agr. Food Chem. 64, 5215–5222. 10.1021/acs.jafc.6b01118 27281292

[B26] Carvajal F. Palma F. Jamilena M. Garrido D. (2015). Preconditioning treatment induces chilling tolerance in zucchini fruit improving different physiological mechanisms against cold injury. Ann. Appl. Biol. 166, 340–354. 10.1111/aab.12189

[B27] Chen G. F. Huo Y. S. Tan D. X. Liang Z. Zhang W. B. Zhang Y. K. (2003). Melatonin in Chinese medicinal herbs. Life Sci. 73, 19–26. 10.1016/s0024-3205(03)00252-2 12726883

[B28] Chen Q. Qi W. B. Reiter R. J. Wei W. Wang B. M. (2009). Exogenously applied melatonin stimulates root growth and raises endogenous indoleacetic acid in roots of etiolated seedlings of *Brassica juncea*. J. Plant. Physiol. 166, 324–328. 10.1016/j.jplph.2008.06.002 18706737

[B29] Dhindsa R. S. Plumb-Dhindsa P. Thorpe T. A. (1981). Leaf senescence: correlated with increased levels of membrane permeability and lipid peroxidation and decreased levels of superoxide dismutase and catalase. J. Exp. Bot. 32, 93–101. 10.1093/jxb/32.1.93

[B30] Ding F. Liu B. Zhang S. X. (2017). Exogenous melatonin ameliorates cold-induced damage in tomato plants. Sci. Hortic. 219, 264–271. 10.1016/j.scienta.2017.03.029

[B31] Dubbels R. Reiter R. J. Klenke E. Goebel A. Schnakenberg E. Ehlers C. (1995). Melatonin in edible plants identified by radioimmunoassay and by high-performance liquid chromatography-mass spectrometry. J. Pineal. Res. 18, 28–31. 10.1111/j.1600-079x.1995.tb00136.x 7776176

[B32] Dumas Y. Dadomo M. Di Lucca G. Grolier P. (2003). Effects of environmental factors and agricultural techniques on antioxidant content of tomatoes. J. Sci. Food Agr. 83 (5), 369–382. 10.1002/jsfa.1370

[B33] Frenkel C. Dyck R. (1973). Auxin inhibition of ripening in Bartlett pears. Plant Physiol. 51, 6–9. 10.1104/pp.51.1.6 16658297 PMC367347

[B34] Fu J. J. Wu Y. Miao Y. J. Xu Y. M. Zhao E. H. Wang J. (2017). Improved cold tolerance in Elymus nutans by exogenous application of melatonin may involve ABA-dependent and ABA- independent pathways. Sci. Rep. 7, 39865. 10.1038/srep39865 28045095 PMC5206618

[B35] Gao H. Lu Z. M. Yang Y. Wang D. N. Yang T. Cao M. M. (2018). Melatonin treatment reduces chilling injury in peach fruit through its regulation of membrane fatty acid contents and phenolic metabolism. Food Chem. 245, 659–666. 10.1016/j.foodchem.2017.10.008 29287423

[B36] Gao H. Zhang Z. K. Chai H. K. Cheng N. Yang Y. Wang D. N. (2016). Melatonin treatment delays postharvest senescence and regulates reactive oxygen species metabolism in peach fruit. Postharvest Bio. Tec. 118, 103–110. 10.1016/j.postharvbio.2016.03.006

[B37] Gong B. Yan Y. Wen D. Shi Q. (2017). Hydrogen peroxide produced by NADPH oxidase: a novel downstream signaling pathway in melatonin-induced stress tolerance in *Solanum lycopersicum*. Physiol. Plantarum. 160, 396–409. 10.1111/ppl12581 28464254

[B38] GonzálezGómez D. Lozano M. FernándezLeón M. F. Ayuso M. C. Bernalte M. J. Rodríguez A. B. (2009). Detection and quantification of melatonin and serotonin in eight sweet cherry cultivars (*Prunus avium L*.). Eur. Food. Res Tec. 229, 223–229. 10.1007/s00217-009-1042-z

[B39] Gu Q. Chen Z. P. Yu X. L. Cui W. T. Pan J. C. Zhao G. (2017). Melatonin confers plant tolerance against cadmium stress via the decrease of cadmium accumulation and reestablishment of microRNA-mediated redox homeostasis. Plant Sci. 261, 28–37. 10.1016/j.plantsci.2017.05.001 28554691

[B40] Hasan M. K. Ahammed G. J. Yin L. L. Shi K. Xia X. J. Zhou Y. H. (2015). Melatonin mitigates cadmium phytotoxicity through modulation of phytochelatins biosynthesis, vacuolar sequestration, and antioxidant potential in *Solanum lycopersicum L*. Front. Plant Sci. 6, 601. 10.3389/fpls.2015.00601 26322055 PMC4531246

[B41] Hattori A. Migitaka H. Iigo M. Itoh M. Yamamoto K. OhtaniKaneko R. (1995). Identification of melatonin in plants and its effects on plasma melatonin levels and binding to melatonin receptors in vertebrates. Biochem. Mol. Bio. Int. 35, 627–634. 7773197

[B42] Hernandez-Ruiz J. Arnao M. B. (2008a). Melatonin stimulates the expansion of etiolated lupin cotyledons. Plant Growth Regul. 55, 29–34. 10.1007/s10725-008-9254-y

[B43] Hernandez-Ruiz J. Arnao M. B. (2008b). Distribution of melatonin in different zones of lupin and barley plants at different ages in the presence and absence of light. J. Agr Food. Chem. 56, 10567–10573. 10.1021/jf8022063 18975965

[B44] Hernandez-Ruiz J. Cano A. Arnao M. B. (2005). Melatonin acts as a growth-stimulating compound in some monocot species. J. Pineal Res. 39, 137–142. 10.1111/j.1600-079X.2005.00226.x 16098090

[B45] Hernandez-Ruiz J. Cano A. Arnao M. B. (2004). Melatonin: growth-stimulating compound present in lupin tissues. Planta 220, 140–144. 10.1007/s00425-004-1317-3 15232696

[B46] Hu W. Kong H. Guo Y. L. Zhang Y. L. Ding Z. H. Tie W. W. (2016). Comparative physiological and transcriptomic analyses reveal the actions of melatonin in the delay of post-harvest physiological deterioration of cassava. Front. Plant Sci. 7, 736. 10.3389/fpls.2016.00736 27303428 PMC4882330

[B47] Hu W. Yang H. Tie W. W. Yan Y. Ding Z. H. Liu Y. (2017). Natural variation in banana varieties highlights the role of melatonin in post-harvest ripening and quality. J. Agr. Food Chem. 65, 9987–9994. 10.1021/acs.jafc.7b03354 29077394

[B48] Huang Y. H. Liu S. J. Yuan S. Guan C. Tian D. Y. Cui X. (2017). Overexpression of ovine *AANAT* and *HIOMT* genes in switchgrass leads to improved growth performance and salt-tolerance. Sci. Rep. 7, 12212. 10.1038/s41598-017-12566-2 28939842 PMC5610178

[B49] Iriti M. Rossoni M. Faoro F. (2006). Melatonin content in grape: Myth or panacea. J. Sci. Food Agric. 86, 1432–1438. 10.1002/jsfa.2537

[B50] Kang K. Lee K. Park S. Kim Y. S. Back K. (2010). Enhanced production of melatonin by ectopic overexpression of human serotonin N-acetyltransferase plays a role in cold resistance in transgenic rice seedlings. J. Pineal Res. 49, 176–182. 10.1111/j.1600-079X.2010.00783.x 20586889

[B51] Kirakosyan A. Seymour E. M. Llanes D. E. U. Kaufman P. B. Bolling S. F. (2009). Chemical profile and antioxidant capacities of tart cherry products. Food Chem. 115, 20–25. 10.1016/j.foodchem.2008.11.042

[B52] Klee H. J. Giovannoni J. J. (2011). Genetics and control of tomato fruit ripening and quality attributes. Ann. Rev. Gene 45, 41. 10.1146/annurev-genet-110410-132507 22060040

[B53] Kocadagli T. Yilmaz C. Gokmen V. (2014). Determination of melatonin and its isomer in foods by liquid chromatography tandem mass spectrometry. Food Chem. 153, 151–156. 10.1016/j.foodchem.2013.12.036 24491714

[B54] Korkmaz A. Değer O. Cuci Y. (2014). Profiling the melatonin content in organs of the pepper plant during different growth stages. Sci. Hortic-Amsterdam 172, 242–247. 10.1016/j.scienta.2014.04.018

[B55] Kuang X. Wang C. Xiang M. Deng L. Deng Y. Q. (2008). The impact of deethylene on Yangmei’s post-harvest physiology and preservation. Chinese Agr. Sci. Bull. 24, 247–251.

[B56] Lee H. Back K. (2017a). Melatonin is required for H2O2- and NO-mediated defense signaling through MAPKKK3 and OXI1 in *Arabidopsis thaliana*. J. Pineal Res. 62, e12379. 10.1111/jpi.12379 27862280

[B57] Lee H. Y. Byeon Y. Back K. (2014). Melatonin as a signal molecule triggering defense responses against pathogen attack in *Arabidopsis* and tobacco. J. Pineal Res. 57, 262–268. 10.1111/jpi.12165 25099383

[B58] Lee H. Y. Byeon Y. Tan D. X. Reiter R. J. Back K. (2015). *Arabidopsis* serotonin N-acetyltransferase knockout mutant plants exhibit decreased melatonin and salicylic acid levels resulting in susceptibility to an avirulent pathogen. J. Pineal Res. 58, 291–299. 10.1111/jpi.12214 25652756

[B59] Lee K. Back K. (2017b). Overexpression of rice serotonin N-acetyltransferase 1 in transgenic rice plants confers resistance to cadmium and senescence and increases grain yield. J. Pineal Res. 62, e12392. 10.1111/jpi12392 28118490

[B60] Li C. Tan D. X. Liang D. Chang C. Jia D. F. Ma F. W. (2015). Melatonin mediates the regulation of ABA metabolism, free-radical scavenging, and stomatal behavior in two Malus species under drought stress. J. Exp. Bot. 66, 669–680. 10.1093/jxb/eru476 25481689

[B61] Li H. Chang J. J. Chen H. J. Wang Z. Y. Gu X. R. Wei C. H. (2017). Exogenous melatonin confers salt stress tolerance to watermelon by improving photosynthesis and redox homeostasis. Front. Plant Sci. 8, 295. 10.3389/fpls.2017.00295 28298921 PMC5331065

[B62] Li H. He J. Yang X. Z. Li X. Luo D. Wei C. H. (2016). Glutathione-dependent induction of local and systemic defense against oxidative stress by exogenous melatonin in cucumber (*Cucumis sativus L*.). J. Pineal. Res. 60, 206–216. 10.1111/jpi.12304 26681257

[B63] Li M. Q. Hasan M. K. Li C. X. Ahammed G. J. Xia X. J. Shi K. (2016b). Melatonin mediates selenium-induced tolerance to cadmium stress in tomato plants. J. Pineal. Res. 61, 291–302. 10.1111/jpi12346 27264631

[B64] Li X. Wei J. P. Scott E. R. Liu J. W. Guo S. Li Y. (2018). Exogenous melatonin alleviates cold stress by promoting antioxidant defense and redox homeostasis in *Camellia sinensis L*. Molecules 23, 165. 10.3390/molecules23010165 PMC6017414 29342935

[B65] Liang C. Z. Zheng G. Y. Li W. Z. Wang Y. Q. Hu B. Wang H. R. (2015). Melatonin delays leaf senescence and enhances salt stress tolerance in rice. J. Pineal. Res. 59, 91–101. 10.1111/jpi.12243 25912474

[B66] Liu C. Chen L. Zhao R. Li R. Zhang S. Yu W. (2019). Melatonin induces disease resistance to *Botrytis cinerea* in tomato fruit by activating jasmonic acid signaling pathway. J. Agric. Food Chem. 67, 6116–6124. 10.1021/acs.jafc.9b00058 31084000

[B67] Liu C. H. Zheng H. H. Sheng K. L. Liu W. Zheng L. (2018). Effects of melatonin treatment on the post-harvest quality of strawberry fruit. Postharvest Bio. Tec. 139, 47–55. 10.1016/j.postharvbio.2018.01.016

[B68] Liu N. Jin Z. Y. Wang S. S. Gong B. A. Wen D. Wang X. F. (2015). Sodic alkaline stress mitigation with exogenous melatonin involves reactive oxygen metabolism and ion homeostasis in tomato. Sci. Hortic-Amsterdam 181, 18–25. 10.1016/j.scienta.2014.10.049

[B69] Ma Q. X. Zhang T. Zhang P. Wang Z. Y. (2016). Melatonin attenuates post-harvest physiological deterioration of cassava storage roots. J. Pineal. Res. 60424-, 434. 10.1111/jpi.12325 26989849

[B70] Manchester L. C. Tan D. X. Reiter R. J. Park W. Monis K. Qi W. B. (2000). High levels of melatonin in the seeds of edible plants: Possible function in germ tissue protection. Life Sci. 67, 3023–3029. 10.1016/s0024-3205(00)00896-1 11125839

[B71] Mercolini L. Mandrioli R. Raggi M. A. (2012). Content of melatonin and other antioxidants in grape-related foodstuffs: measurement using a MEPS-HPLC-F method. J. Pineal. Res. 53, 21–28. 10.1111/j.1600-079x.2011.00967.x 22017461

[B72] Murch S. J. Hall B. A. Le C. H. Saxena P. K. (2010). Changes in the levels of indoleamine phytochemicals during véraison and ripening of wine grapes. J. Pineal. Res. 49, 95–100. 10.1111/j.1600-079x.2010.00774.x 20536685

[B73] Muszynska B. Kala K. Sulkowska-Ziaja K. Krakowska A. Opoka W. (2016). *Agaricus bisporus* and its in vitro culture as a source of indole compounds released into artificial digestive juices. Food Chem. 199, 509–515. 10.1016/j.foodchem.2015.12.041 26776002

[B74] Muszynska B. Sulkowska-Ziaja K. (2012). Analysis of indole compounds in edible Basidiomycota species after thermal processing. Food Chem. 132455-, 459. 10.1016/j.foodchem.2011.11.021 26434315

[B75] Mythili R. S. Sathiavelu A. (2010). Recovery of bacteriocin (nisin) from *Lactococcus lactis* and testing its ability to increase the shelf life of vegetables (carrot and beans). Res. J. Biol. Sci. 5, 727–730. 10.3923/rjbsci.2010.727.730

[B76] Nawaz M. A. Jiao Y. Y. Chen C. Shireen F. Zheng Z. H. Imtiaz M. (2018). Melatonin pretreatment improves vanadium stress tolerance of watermelon seedlings by reducing vanadium concentration in the leaves and regulating melatonin biosynthesis and antioxidant-related gene expression. J. Plant Physiol. 220, 115–127. 10.1016/j.jplph.2017.11.003 29172132

[B77] Ni J. Wang Q. J. Shah F. A. Liu W. B. Wang D. D. Huang S. W. (2018). Exogenous melatonin confers cadmium tolerance by counterbalancing the hydrogen peroxide homeostasis in wheat seedlings. Molecules 23, 799. 10.3390/molecules23040799 PMC6017192 29601513

[B78] Okazaki M. Ezura H. (2009). Profiling of melatonin in the model tomato (*Solanum lycopersicum L*.) cultivar Micro-Tom. J. Pineal Res. 46, 338–343. 10.1111/j.1600-079X.2009.00668.x 19317796

[B79] Palma F. Carvajal F. Ramos J. M. Jamilena M. Garrido D. (2015). Effect of putrescine application on maintenance of zucchini fruit quality during cold storage: contribution of GABA shunt and other related nitrogen metabolites. Postharvest Bio. Tec. 99, 131–140. 10.1016/j.postharvbio.2014.08.010

[B80] Pennycooke J. C. Cox S. Stushnoff C. (2005). Relationship of cold acclimation, total phenolic content and antioxidant capacity with chilling tolerance in petunia (*Petunia × hybrida*). Environ. Exp. Bot. 53, 225–232. 10.1016/j.envexpbot.2004.04.002

[B81] Prasanna V. Prabha T. N. Tharanathan R. N. (2007). Fruit ripening phenomena—an overview. Crit. Rev. Food Sci. 47, 1–19. 10.1080/10408390600976841 17364693

[B82] Puerta-Gomez A. F. Cisneros-Zevallos L. (2011). Postharvest studies beyond fresh market eating quality: phytochemical antioxidant changes in peach and plum fruit during ripening and advanced senescence. Postharvest Biol. Tec. 60, 220–224. 10.1016/j.postharvbio.2011.01.005

[B83] Qi Z. Y. Wang K. X. Yan M. Y. Kanwar M. K. Li D. Y. Wijaya L. (2018). Melatonin alleviates high temperature-induced pollen abortion in *Solanum lycopersicum*. Molecules 23, 386. 10.3390/molecules23020386 PMC6017144 29439470

[B84] Qian Y. Q. Tan D. X. Reiter R. J. Shi H. T. (2015). Comparative metabolomic analysis highlights the involvement of sugars and glycerol in melatonin-mediated innate immunity against bacterial pathogen in *Arabidopsis*. Sci. Rep. 5, 15815. 10.1038/srep15815 26508076 PMC4623600

[B85] Reiter R. J. Tan D. X. Cabrera J. D’Arpa D. Sainz R. M. Mayo J. C. (1999). The oxidant/antioxidant network: Role of melatonin. Biol. Signals Recept. 8, 56–63. 10.1159/000014569 10085463

[B86] Riga P. Medina S. García-Flores L. A. Gil-Izquierdo Á. (2014). Melatonin content of pepper and tomato fruits: effects of cultivar and solar radiation. Food Chem. 156, 347–352. 10.1016/j.foodchem.2014.01.117 24629979

[B87] Rugkong A. McQuinn R. Giovannoni J. J. Rose J. K. C. Watkins C. B. (2011). Expression of ripening-related genes in cold-stored tomato fruit. Postharvest. Biol. Tec. 61, 1–14. 10.1016/j.postharvbio.2011.02.009

[B88] Rui W. Yang X. L. Xu H. Li T. L. (2016). Research progress of melatonin biosynthesis and metabolism in higher plants. Plant Physiol. J. 52, 615–627. 10.13592/j.cnki.ppj.2016.0052

[B89] Shewfelt R. L. del Rosario B. A. (2000). The role of lipid peroxidation in storage disorders of fresh fruits and vegetables. Hortsci. Publ. of the Am. Soc. Hort. Sci. 35, 575–579. 10.21273/HORTSCI.35.4.575

[B90] Shi H. T. Qian Y. Q. Tan D. X. Reiter R. J. He C. (2015a). Melatonin induces the transcripts of CBF/DREB1s and their involvement in both abiotic and biotic stresses in *Arabidopsis*. J. Pineal Res. 59, 334–342. 10.1111/jpi.12262 26182834

[B91] Shi H. T. Chen Y. H. Tan D. X. Reiter R. J. Chan Z. L. He C. Z. (2015b). Melatonin induces nitric oxide and the potential mechanisms relate to innate immunity against bacterial pathogen infection in *Arabidopsis*. J. Pineal Res. 59, 102–108. 10.1111/jpi.12244 25960153

[B92] Shi H. T. Wei Y. X. He C. Z. (2016). Melatonin-induced CBF/DREB1s are essential for diurnal change of disease resistance and CCA1 expression in *Arabidopsis*. Plant Physiol. Bioch. 100, 150–155. 10.1016/j.plaphy.2016.01.018 26828406

[B93] Simopoulos A. P. Tan D. X. Manchester L. C. Reiter R. J. (2005). Purslane: A plant source of omega-3 fatty acids and melatonin. J. Pineal Res. 39, 331–332. 10.1111/j.1600-079X.2005.00269.x 16150116

[B94] Song L. L. Wang J. H. Shafi M. Liu Y. Wang J. Wu J. S. (2016). Hypobaric treatment effects on chilling injury, mitochondrial dysfunction, and the ascorbate-glutathione (ASA-GSH) cycle in postharvest peach fruit. J. Agric. Food. Chem. 64, 4665–4674. 10.1021/acs.jafc.6b00623 27195461

[B95] Stege P. W. Sombra L. L. Messina G. Martinez L. D. Silya M. F. (2010). Determination of melatonin in wine and plant extracts by capillary electrochromatography with immobilized carboxylic multi-walled carbon nanotubes as stationary phase. Electrophoresis 1, 2242–2248. 10.1002/elps.200900782 20593400

[B96] Stürtz M. Cerezo A. B. Cantos-Villar E. Garcia-Parrilla M. C. (2011). Determination of the melatonin content of different varieties of tomatoes (*Lycopersicon esculentum*) and strawberries (*Fragaria ananassa*). Food Chem. 127 (3), 1329–1334. 10.1016/j.foodchem.2011.01.093 25214134

[B97] Sun Q. Q. Zhang N. Wang J. F. Cao Y. Y. Li X. S. Zhang H. J. (2016). A label-free differential proteomics analysis reveals the effect of melatonin in promoting fruit ripening and anthocyanin accumulation upon post-harvest in tomatoes. J. Pineal. Res. 61, 138–153. 10.1111/jpi.12315 26820691

[B98] Sun Q. Q. Zhang N. Wang J. F. Zhang H. J. Li D. B. Shi J. (2015). Melatonin promotes ripening and improves quality of tomato fruit during postharvest life. J. Exp. Bot. 66, 657–668. 10.1093/jxb/eru332 25147270 PMC4321535

[B99] Tan D. X. Manchester L. C. Liu X. Y. Rosales-Corral S. A. Acuna-Castroviejo D. Reiter R. J. (2013). Mitochondria and chloroplasts as the original sites of melatonin synthesis: a hypothesis related to melatonin’s primary function and evolution in eukaryotes. J. Pineal. Res. 54, 127–138. 10.1111/jpi.12026 23137057

[B100] Tan D. X. Reiter R. J. Manchester L. C. Yan M. T. El-Sawi M. Sainz R. M. (2002). Chemical and physical properties and potential mechanisms: melatonin as a broad spectrum antioxidant and free radical scavenger. Curr. Top. Med. Chem. 2, 181–197. 10.2174/1568026023394443 11899100

[B101] Tingwa P. O. Young R. E. (1975). The effect of indole-3-acetic acid and other growth regulators on the ripening of avocado fruits. Plant Physiol. 55, 937–940. 10.1104/pp.55.5.937 16659195 PMC541737

[B102] Vendrell M. (1969). Reversion of senescence: effects of 2,4-dichlorophenoxyacetic acid and indoleacetic acid on respiration, ethylene production, and ripening of banana fruit slices. Aust. J. Biol. Sci. 22, 601–610. 10.1071/bi9690601

[B103] Verde A. Míguez J. M. Gallardo M. (2018). Melatonin and related bioactive compounds in commercialized date palm fruits (*Phoenix dactylifera L*.): correlation with some antioxidant parameters. Eur Food Res Technol. 245, 51–59. 10.1007/s00217-018-3139-8

[B104] Vitalini S. Gardana C. Zanzotto A. Simonetti P. Faoro F. Fico G. (2011). The presence of melatonin in grapevine (*Vitis vinifera L*.) berry tissues. J. Pineal. Res. 51, 331–337. 10.1111/j.1600-079X.2011.00893.x 21615489

[B105] Vogt W. (1995). Oxidation of methionyl residues in proteins: tools, targets, and reversal. Free Radical Bio. Med. 18, 93–105. 10.1016/0891-5849(94)00158-G 7896176

[B106] Wang C. Yin L. Y. Shi X. Y. Xiao H. Kang K. Liu X. Y. (2016a). Effect of cultivar, temperature, and environmental conditions on the dynamic change of melatonin in mulberry fruit development and wine fermentation. J. Food Sci. 81, M958–M967. 10.1111/1750-384113263 26953927

[B107] Wang J. Z. Zhang S. L. Zhou Q. Tao S. T. Shahrokh K. Ye Y. X. (2012). The effect of six post-harvest treatment methods on strawberry fruit quality. Food Res. Dev. 33, 179–181.

[B108] Wang L. Feng C. Zheng X. D. Guo Y. Zhou F. F. Shan D. Q. (2017). Plant mitochondria synthesize melatonin and enhance the tolerance of plants to drought stress. J. Pineal. Res. 63, e12429. 10.1111/jpi.12429 28599069

[B109] Wang L. Zhao Y. Reiter R. J. He C. J. Liu G. S. Lei Q. (2014). Changes in melatonin levels in transgenic ‘Micro-Tom’ tomato overexpressing ovine AANAT and ovine HIOMT genes. J. Pineal Res. 56, 134–142. 10.1111/jpi.12105 24138427

[B110] Wang Q. N. An B. Wei Y. X. Reiter R. J. Shi H. T. Luo H. T. (2016b). Melatonin regulates root meristem by repressing auxin synthesis and polar auxin transport in *Arabidopsis*. Front Plant Sci. 7, 1882. 10.3389/fpls.201601882 28018411 PMC5156734

[B111] Wang X. Wang Y. Z. Liu G. S. Liu C. L. Li P. H. (2008). Effects of IAA, GA and ABA on the Activities of Ca∼(2+)-ATPase in Variety ‘Hongdeng’ Sweet cherry Fruit. J. Qingdao Agr. Univ. 25, 88–90.

[B112] Wei Y. X. Zeng H. Q. Hu W. Chen L. Z. He C. Z. Shi H. T. (2016). Comparative transcriptional profiling of melatonin synthesis and catabolic genes indicates the possible role of melatonin in developmental and stress responses in rice. Front. Plant. Sci. 7, 676. 10.3389/fpls.2016.00676 27242875 PMC4870392

[B113] Wen D. Gong B. A. Sun S. S. Liu S. Q. Wang X. F. Wei M. (2016). Promoting roles of melatonin in adventitious root development of *Solanum lycopersicum L*. by regulating auxin and nitric oxide signaling. Front. Plant Sci. 7, 718. 10.3389/fpls.2016.00718 27252731 PMC4879336

[B114] Xin D. D. Si J. J. Kou L.,. P. (2017). Post-harvest exogenous melatonin enhances quality and delays the senescence of cucumber. Acta Hortic. Sin. 44, 891–901. 10.16420/j.issn.0513-353x.2016-0888

[B115] Xu L. Yue Q. Bian F. Sun H. Zhai H. Yao Y. (2017). Melatonin enhances phenolics accumulation partially via ethylene signaling and resulted in high antioxidant capacity in grape berries. Front. Plant Sci. 8, 1426. 10.3389/fpls.2017.01426 28868058 PMC5563355

[B116] Xu W. Cai S. Y. Zhang Y. Wang Y. Ahammed G. J. Xia X. J. (2016). Melatonin enhances thermotolerance by promoting cellular protein protection in tomato plants. J Pineal Res. 61, 457–469. 10.1111/jpi.12359 27484733

[B117] Xu X. D. Sun Y. Sun B. Zhang J. Guo X. Q. (2010). Effects of exogenous melatonin on active oxygen metabolism of cucumber seedlings under high temperature stress. Chinese J. Appl. Ecol. 21, 1295–1300. 10.3724/SP.J.1142.2010.40521 20707116

[B118] Yang W. J. Du Y. T. Zhou Y. B. Chen J. Xu Z. S. Ma Y. Z. (2019). Overexpression of TaCOMT improves melatonin production and enhances drought tolerance in transgenic *Arabidopsis*. Int. J. Mol. Sci. 20, 652. 10.3390/ijms20030652 PMC6387377 30717398

[B119] Yin L. H. Wang P. Li M. J. Ke X. W. Li C. Y. Liang D. (2013). Exogenous melatonin improves Malus resistance to Marssonina apple blotch. J. Pineal Res. 54, 426–434. 10.1111/jpi.12038 23356947

[B120] Yu K. D. Zhu K. L. Ye M. J. Zhao Y. P. Chen W. R. Guo W. D. (2016). Heat tolerance of highbush blueberry is related to the antioxidative enzymes and oxidative protein-repairing enzymes. Sci. Hortic-Amsterdam 198, 36–43. 10.1016/j.scienta.2015.11.018

[B121] Zhai R. Liu J. L. Liu F. X. Zhao Y. X. Liu L. L. Fang C. (2018). Melatonin limited ethylene production, softening and reduced physiology disorder in pear (*Pyrus communis, L*.) fruit during senescence. Postharvest Bio. Tec. 139, 38–46. 10.1016/j.postharvbio.2018.01.017

[B122] Zhang H. J. Zhang N. Yang R. C. Wang L. Sun Q. Q. Li D. B. (2014). Melatonin promotes seed germination under high salinity by regulating antioxidant systems, ABA and GA4 interaction in cucumber (*Cucumis sativus L*.). J. Pineal Res. 57, 269–279. 10.1111/jpi.12167 25112973

[B123] Zhang H. X. Liu X. Chen T. Ji Y. X. Shi K. Wang L. (2018a). Melatonin in apples and juice: inhibition of browning and microorganism growth in apple juice. Molecules 23, 521. 10.3390/molecules23030521 PMC6017754 29495435

[B124] Zhang J. Shi Y. Zhang X. Z. Du H. M. Xu B. Huang B. R. (2017). Melatonin suppression of heat-induced leaf senescence involves changes in abscisic acid and cytokinin biosynthesis and signaling pathways in perennial ryegrass (*Lolium perenne L*.). Environ. Exp. Bot. 138, 36–45. 10.1016/j.envexpbot.2017.02.012

[B125] Zhang N. Zhang H. J. Zhao B. Sun Q. Q. Cao Y. Y. Li R. (2014b). The RNA-seq approach to discriminate gene expression profiles in response to melatonin on cucumber lateral root formation. J. Pineal Res. 56, 39–50. 10.1111/jpi.12095 24102657

[B126] Zhang N. Zhao B. Zhang H. J. Weeda S. Yang C. Yang Z. C. (2012). Melatonin promotes water-stress tolerance, lateral root formation, and seed germination in cucumber (*Cucumis sativus L*.). J. Pineal. Res. 54, 15–23. 10.1111/j.1600-079X.2012.01015.x 22747917

[B127] Zhang S. M. Zheng X. Z. Reiter R. J. Feng S. Wang Y. Liu S. (2017b). Melatonin attenuates potato late blight by disrupting cell growth, stress tolerance, fungicide susceptibility and homeostasis of gene expression in *Phytophthora infestans*. Front. Plant Sci. 8, 1993. 10.3389/fpls.2017.01993 29209352 PMC5702310

[B128] Zhang Y. Y. Huber D. J. Hu M. J. Jiang G. X. Gao Z. Y. Xu X. B. (2018b). Melatonin delays postharvest browning in litchi fruit by enhancing anti-oxidative processes and oxidation repair. J. Agr. Food Chem. 66, 7475–7484. 10.1021/acs.jafc8b01922 29953220

[B129] Zhao H. L. Ye L. Wang Y. P. Zhou X. T. Yang J. W. Wan J. W. (2016). Melatonin increases the chilling tolerance of chloroplast in cucumber seedlings by regulating photosynthetic electron fux and the Ascorbate-Glutathione cycle. Front. Plant Sci. 7, 1314. 10.3389/fpls.2016.01814 27999581 PMC5138187

[B130] Zhao Y. Tan D. X. Lei Q. Chen H. Wang L. Li Q. T. (2013). Melatonin and its potential biological functions in the fruits of sweet cherry. J. Pineal. Res. 55, 79–88. 10.1111/jpi.12044 23480341

[B131] Zhou H. W. Lurie S. Lers A. Khatchitski A. Sonego L. Arie R. B. (2000). Delayed storage and controlled atmosphere storage of nectarines: two strategies to prevent woolliness. Postharvest Biol. Tec. 18, 133–141. 10.1016/S0925-5214(99)00072-1

[B132] Zhu L. L. Hu H. L. Luo S. F. Wu Z. X. Li P. X. (2018). Melatonin delaying senescence of postharvest broccoli by regulating respiratory metabolism and antioxidant activity. *T*. Chinese. Soc. Agr. Eng. 34, 300–308.

[B133] Zhu Z. Q. Lee B. (2015). Friends or foes: new insights in jasmonate and ethylene co-actions. Plant Cell Physiol. 56, 414–420. 10.1093/pcp/pcu171 25435545

[B134] Zuo B. X. Zheng X. D. He P. L. Wang L. Lei Q. Feng C. (2014). Overexpression of MzASMT improves melatonin production and enhances drought tolerance in transgenic *Arabidopsis thaliana* plants. J. Pineal Res. 57, 408–417. 10.1111/jpi.12180 25250844

